# Cultural and linguistic diversity in dementia: data from an Australian memory clinic

**DOI:** 10.1002/alz.088391

**Published:** 2025-01-09

**Authors:** Kai Sin Chin

**Affiliations:** ^1^ Royal Melbourne Hospital, Melbourne, VIC, Australia; The Walter and Eliza Hall Institute of Medical Research, Parkville, VIC Australia

## Abstract

**Background:**

Australia has a rich migration history, with one in three older people coming from a culturally and linguistically diverse (CALD) background. Patients from CALD backgrounds tend to present at later stages of their diseases but face difficulties accessing appropriate dementia care compared to English‐speaking patients. Limited literature exists on the clinical experience among CALD patients who have been historically underrepresented in dementia research. This study therefore aimed to investigate and describe the clinical characteristics and service provision for CALD patients presenting to the Memory Clinic at the Royal Melbourne Hospital, a tertiary hospital which serves a significant proportion of CALD patients.

**Method:**

A retrospective analysis was conducted. All consecutive patients who presented to the Memory Clinic between September 2016 and June 2018 were included. Data collected included patient demographics, medical history, cognitive test scores, neuropsychiatric symptoms, other clinical scales, clinical diagnoses, and length of follow up.

**Result:**

A total of 301 patients (mean age 78.8 ± 8.2; 55.8% female) were included and 48.5% were from a CALD background, the majority being Italian. Patients from CALD backgrounds were more likely to be living with family (73.3% vs 57.4%, OR [odds ratio] 2.1) and less likely to have attended education beyond primary level (26.7% vs 83.9%, OR 14.3). Cognitively, CALD patients had poorer performance on MMSE (median MMSE 18 vs 25) and were more likely to be diagnosed with dementia (46.6% vs 31.0%, OR 1.9) at their initial assessments. Additionally, CALD patients were less likely to be referred for neuropsychology (28.8% vs 50.3%, OR 0.40).

**Conclusion:**

The study findings highlight the differences in clinical characteristics as well as inherent issue of inequality in healthcare among patients from CALD backgrounds in an Australian clinical setting. Given the recent approval of disease‐modifying anti‐amyloid therapy in mild Alzheimer’s disease, more research is warranted to help diagnose dementia at earlier stages in CALD patients and ultimately promote more equitable and culturally inclusive dementia care.

